# Identification of somatic mutations in monozygotic twins discordant for psychiatric disorders

**DOI:** 10.1038/s41537-018-0049-5

**Published:** 2018-04-13

**Authors:** Masaki Nishioka, Miki Bundo, Junko Ueda, Akane Yoshikawa, Fumichika Nishimura, Tsukasa Sasaki, Chihiro Kakiuchi, Kiyoto Kasai, Tadafumi Kato, Kazuya Iwamoto

**Affiliations:** 1 Department of Molecular Psychiatry, Graduate School of Medicine, The University of Tokyo, Tokyo, Japan; 2 Department of Neuropsychiatry, Graduate School of Medicine, The University of Tokyo, Tokyo, Japan; 3 Division for Counseling and Support, The University of Tokyo, Tokyo, Japan; 4 Department of Molecular Brain Science, Graduate School of Medical Sciences, Kumamoto University, Kumamoto, Japan; 5 PRESTO, Japan Science and Technology Agency, Saitama, Japan; 6 Laboratory for Molecular Dynamics of Mental Disorders, RIKEN Brain Science Institute, Saitama, Japan; 7 Department of Physical and Health Education, Graduate School of Education, The University of Tokyo, Tokyo, Japan

## Abstract

Monozygotic twins are assumed to have identical genomes. Based on this assumption, phenotypic discordance in monozygotic twins has been previously attributed to environmental factors. However, recent genomic studies have identified characteristic somatic mutations in monozygotic twins discordant for Darier disease, Van der Woude syndrome, and Dravet syndrome. Here, we explored somatic mutations in four pairs of monozygotic twins discordant for schizophrenia or delusional disorder. We analyzed whole exome sequence data obtained from blood samples and identified seven somatic mutations in one twin pair discordant for delusional disorder. All seven of these mutations were validated by independent amplicon sequencing, and five of them were further validated by pyrosequencing. One somatic mutation in the patient with delusional disorder showed a missense variant in *ABCC9* with an allele fraction of 7.32%. Although an association between the somatic mutations and phenotypic discordance could not be established conclusively in this study, our results suggest that somatic mutations in monozygotic twins may contribute to the development of psychiatric disorders, and can serve as high-priority candidates for genetic studies.

## Genetics: Identical twins can still differ genetically, with impacts on psychosis

Identical twins are not always identical when it comes to psychiatric disorders—and DNA mutations that arise after birth could explain why. Researchers in Japan led by Tadafumi Kato from the RIKEN Brain Science Institute and  Kazuya Iwamoto from Kumamoto University searched for DNA differences between four pairs of identical twins discordant for schizophrenia or delusional disorder by sequencing the entire protein-coding portion of the genome from the study subjects’ blood. In one sibling pair, they found seven genetic differences, including one in the sister with the delusional disorder that altered the sequence of a protein implicated in sleep and other brain functions. The findings suggest that, alongside epigenetic and environmental differences, acquired mutations can account for discordances in psychiatric illnesses among otherwise genetically identical twins.

## Introduction

Monozygotic (MZ) twins are assumed to have identical genomes. Based on this assumption, phenotypic discordance between MZ twins has been attributed to environmental factors. However, recent genomic studies have discovered several cases of healthy MZ twins who have discordant mutations.^1,2^ Considering MZ twins have identical genomes at the time of fertilization, these mutations must have arisen in the soma after fertilization. In addition, several cases have been discovered where MZ twins discordant for some rare disease exhibited discordant mutations in peripheral tissue samples. In each of the following cases, only the affected twin, not the healthy co-twin, exhibited pathogenic mutations: in *ATP2A2* for Darier disease,^3^ in *IRF6* for Van der Woude syndrome,^4^ in *SCN1A* for Dravet syndrome,^5^ and in *NF1* for neurofibromatosis type 1.^6^ Although these somatic mutations were identified in peripheral tissues, they must have been shared among various tissues and were therefore pathogenic for the patients. Although not conclusive regarding the effect on the phenotype, one study that carried out exome-wide investigation detected mutations in *FBXO38*, *SMOC2*, and *TDRP* only in the affected twin in a pair of MZ twins discordant for gender dysphoria.^7^

In parallel with the findings in MZ twins, recent genomic studies have revealed that different somatic cells have different mutational profiles in the same individual.^8,9^ Some somatic mutations can cause dysfunction in the affected organs, resulting in phenotypic variation. In fact, somatic mutations in critical genes in patients with neuropsychiatric diseases have been reported; single nucleotide variants (SNVs) on *AKT3*, *PIKCA3*, and *MTOR* in the brains of patients with hemimegalencephaly and cortical dysplasia,^10–13^ SNVs on *CACNA1C*, *SCN1A*, and *SETD2* in the brains of patients with autism spectrum disorder (ASD) and fragile X syndrome,^14^ and increased LINE-1 copy number in the brains of patients with Rett syndrome, ataxia-telangiectasia and schizophrenia.^15–17^ These studies suggested possible links between somatic mutations and psychiatric disorders, as well as brain malformation diseases.

Somatic mutations in the known risk genes for neuropsychiatric diseases have also been reported in samples of peripheral tissues. SNVs were found in *PIK3CA* in the blood and saliva of patients with hemimegalencephaly,^18^ and in *DCX* and *LIS1* in the blood samples of patients with double-cortex syndrome, polymicrogyria with megalencephaly, periventricular nodular heterotopia, and pachygyria.^19^ Somatic mutations in *MECP2* were reported in the blood samples of patients with Rett syndrome.^20^ Four groups analyzed whole exome sequence (WES) data from the Simons Simplex Collection (a collection of large cohorts of patients with ASD) and identified several hundred somatic mutations in blood samples, including those in known risk genes, such as *CHD2*, *RELN*, *SCN2A*, *SYNGAP*, and other genes.^21–24^ Sequencing of blood samples of patients with ASD showed frequent mutations in critical exons of genes expressed during early brain development, especially genes expressed in the prenatal amygdala.^23^ The contribution of these somatic mutations to the diagnosis was estimated to be 3–5%.^21,22,24^ Somatic mutations with large allele fractions should occur early in development, shared among multiple tissues including the brain and blood. The relevance of the identified genes implicates that these mutations were probably shared by the brain and had a pathogenic role. Somatic mutations are one candidate explanation for neuropsychiatric disorders as rare variants, in addition to germline rare variants, which have been extensively elucidated in ASD and schizophrenia.^25–32^

Here, we discovered somatic mutations by WES data of peripheral blood cells derived from MZ twins discordant for schizophrenia or delusional disorder. The candidate mutations underwent multi-layered filtering and rigorous validation by ultra-deep target amplicon sequencing (TAS). We found that one MZ twin pair discordant for delusional disorder carried a few somatic SNVs including one missense variant in *ABCC9*. Although an association between the somatic mutations and phenotypic discordance between MZ twins could not be established conclusively, our results suggest that somatic mutations might be related to the development of psychiatric disorders in MZ twins. Further, somatic mutations discovered in MZ twins can serve as high-priority candidates for genetic studies.

## Results

### Sequencing and quality control (QC)

We performed WES of genomic DNA isolated from blood samples obtained from four pairs of MZ twins discordant for schizophrenia or delusional disorder, and obtained sequence data at depth of 150.5× on average (Table 1). Somatic mutations in MZ twins were detected from WES data and then validated by TAS and pyrosequencing. After QC to optimize the sequence quality and depth for somatic mutation detection, the average read depth decreased drastically from 150.5 to 82.2. We stringently eliminated low-quality base-calls to preclude false positives from the sequencing errors. After QC, we achieved a coverage of 85.1% on average with respect to the target exome with a depth ≥30.

**Table 1:** Summary of sample and sequence data

Sample data					Sequence data							
ID	Sample	Sex	Age	Psychiatric disorder	Region	Read length	Read pair	Raw DP	QC DP	10 × Cov.	30 × Cov.	60 × Cov.
FT11^a^	Blood	Female	40s	Schizophrenia	V4 + UTR	100	53940007	153.3	92.1	97.9	86.2	64.6
FT12	Blood	Female	40s	None	V4 + UTR	100	59159933	168.1	99.7	98.3	88.0	64.6
JT11^a^	Blood	Female	40s	Schizophrenia	V5 + UTR	100	54100195	145.1	74.8	97.5	84.2	50.0
JT12	Blood	Female	40s	None	V5 + UTR	100	60187660	161.4	85.4	97.8	87.8	58.0
TT21^a^	Blood	Female	20s	Schizophrenia	V5 + UTR	100	43965857	117.9	64.0	96.8	77.4	40.2
TT22	Blood	Female	20s	None	V5 + UTR	100	48817259	130.9	69.4	97.3	81.2	45.0
TT11^a^	Blood	Female	60s	Delusional Disorder	V5 + UTR	100	56239238	150.8	79.9	97.7	86.2	54.0
TT12	Blood	Female	60s	None	V5 + UTR	100	65675687	176.1	92.5	98.0	89.8	62.9

Agilent SureSelect Human All Exon V4 + UTR was used for FT11 and FT12, and V5 + UTR was used for the other twin samples. Only approximate ages of the subjects have been provided to protect their privacy

DP: average depth of alignment data, QC DP: DP after quality control, Cov.: percentage of target exome covered by quality controlled alignment data, based on only nonN bases

^a^Indicates patient

The genotype concordance rates between the co-twins FT11 and FT12, JT11 and JT12, TT21 and TT22, and TT11 and TT12 were all 100% at 1278, 1097, 998, and 1042 putatively credible SNV sites, respectively. To select putatively credible SNV sites for obtaining a rough estimate of monozygosity, we used only three parameters: the depth, genotype quality, and GATK-defined QUAL score. The genotype concordance confirmed the monozygosity of the four pairs.

### Detection and validation of somatic mutations in MZ twins

We ran MuTect on all four pairs of MZ twins. It detected 98.5 somatic SNV candidates on average in each sample (Supplementary Table S1). After applying our filtering parameters to the somatic mutation candidates, we obtained 28 high-confidence (HC; defined in the methods section) candidate sites in total (Supplementary Tables S1). We performed TAS on these 28 HC candidate sites, validating seven of them (Table 2 and Supplementary Tables S2). They all hailed from a single pair of MZ twins discordant for delusional disorder. The allele fractions of seven somatic mutations in the twins were found to range from 1.12 to 7.32% upon TAS. The allele fractions of these seven somatic mutations in the control co-twin were found to be 0% upon WES and far below the assumed error rate (0.316%) in TAS.

**Table 2:** TAS-validated somatic mutations with corresponding allele fractions and functional annotation

Chr	Position	Ref	Alt	Allele fraction (%)						Gene	SO	HGVS.p
				WES		TAS		Pyrosequence				
				TT11^a^	TT12	TT11^a^	TT12	TT11^a^	TT12			
7	105641974	G	T	4.3	0	2.417	0.012	0	0	*CDHR3*	synonymous_variant	p.Ala260Ala
11	72947061	C	T	9.2	0	5.770	0.010	11	0	*P2RY2*	3_prime_UTR_variant	
12	22040794	A	C	8.1	0	7.320	0.007	11	0	*ABCC9*	missense_variant	p.Leu626Arg
1	21605869	G	A	0	6.3	0.014	3.830	0	5	*ECE1*	missense_variant	p.Pro20Leu
1	39991592	C	T	0	11.3	0.006	6.588	0	8	*BMP8A*	3_prime_UTR_variant	
1	245849059	C	T	0	5.5	0.012	1.120	0	0	*KIF26B*	missense_variant	p.Thr925Met
12	78571018	C	T	0	5.8	0.016	3.092	0	3	*NAV3*	missense_variant	p.Pro1741Leu

SO: Sequence ontology categories defined by the Sequence Ontology project (http://www.sequenceontology.org/). HGVS.p: Amino acid change described according to the Human Genome Variation Society

^a^Indicates patient (with delusional disorder in this table). After TAS, the allele fractions of controls were found to be <0.316% (the assumed error rate). The genomic positions are based on the human reference genome (GRCh37)

### Detection of somatic mutation by Strelka

Strelka and further filtering procedures yielded 22 HC candidates (Supplementary Tables S1), five of which were validated by TAS (Supplementary Tables S2). Hence, the validation rates for Strelka and MuTect were 5/22 (22.7%) and 7/28 (25%), respectively. All five TAS-validated candidates by Strelka were also detected by MuTect. Therefore, MuTect seemed to be superior to Strelka with respect to both sensitivity and specificity. The combined validation rate for MuTect and Strelka was 5/7 (71.4%).

### Pyrosequencing validation

**Fig. 1:**
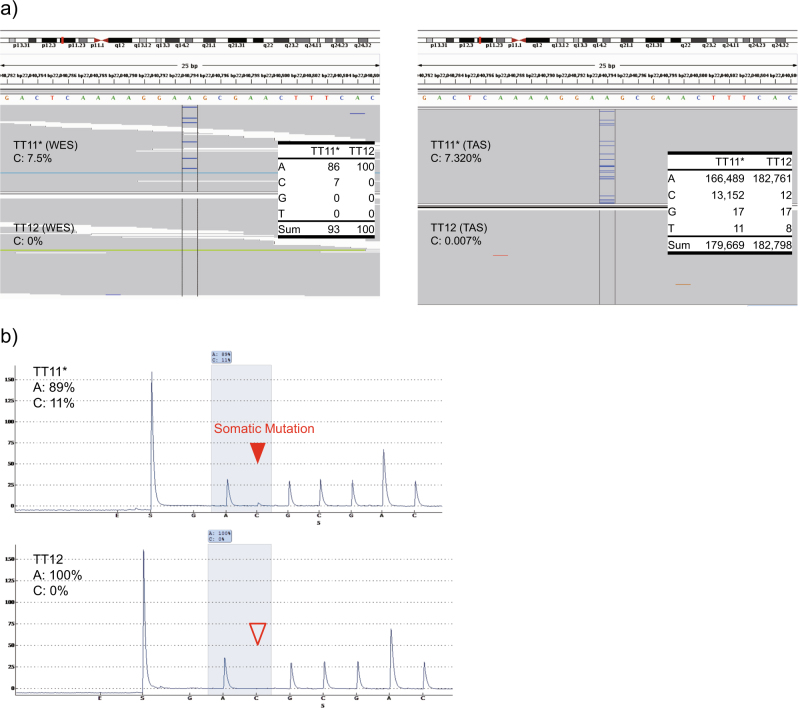
Visualization of the validated somatic mutation in *ABCC9.* **a** The WES (left) and TAS (right) alignments encompassing the chr12:22040794 site of *ABCC9* visualized by IGV. WES data shows that seven alternate alleles (C) were detected in place of the reference allele (A) in TT11; the WES data of TT12 shows that no C alleles were observed. We observed 13152 C alleles in place of the reference A allele (7.32%, depth = 179669×) in TT11 in TAS. In contrast, we detected 12C alleles (0.007%, depth = 182798×) in TT12 in TAS. The latter was a much smaller fraction than the assumed sequencing error rate of 0.316%. **b** Pyrograms of site chr12:22040794 (A > C) in *ABCC9* are shown. The somatic mutation (C allele) was detected in the twin with delusional disorder, TT11 (allele fraction 11%), but not detected in the co-twin without the disorder, TT12. E and S denote enzyme and substrate during pyrosequencing

We performed further validation by pyrosequencing the seven TAS-validated mutations. In accordance with the previously reported sensitivity of pyrosequencing,^33^ pyrosequencing validated the five HC sites whose alternate allele fraction (AAF) came out above 3% upon TAS (Table 2). The somatic mutation in *ABCC9* was also validated by pyrosequencing (Fig. 1). Allele fractions of the five validated somatic mutations were found to be 0% in the control co-twin upon pyrosequencing.

### Post hoc analysis of TAS-validated mutations

We investigated differences in the values of the filtering parameters (base quality (BQ) and depth (DP)) between the validated and unvalidated HC candidates detected by MuTect (Supplementary Table S2). The BQ of the seven TAS-validated and 21 unvalidated HC candidates was not significantly different (one-sided *t*-test *p*-value = 0.201). The average BQ values of TAS-validated and unvalidated candidates were 33.4 ± 2.0 (standard deviation) and 32.9 ± 1.1, respectively. The average base qualities were more than 30 in both groups in this analysis.

The DP of the seven TAS-validated and 21 unvalidated HC candidates was significantly different (one-sided *t*-test *p*-value = 4.318 × 10^−7^). The average DP values of the TAS-validated and unvalidated candidates were 81.6 ± 14.0 and 44.2 ± 12.5, respectively. A DP threshold > 60 showed a sensitivity of 100% and a specificity of 90.5% in selecting true positive somatic mutations. The average coverage with a depth of over 60× was 54.9% (Table 1), which means half of the target regions were not sequenced deep enough to reliably detect somatic mutations. The BLAT score of the seven sites that were validated by TAS was ≤32 (Supplementary Table S2).

### Predicted functional effects of the validated mutations

Among the seven TAS-validated HC sites, four were missense variants, including one in *ABCC9* (ATP-binding cassette, sub-family C, member 9) with an AAF of 7.32% in the patient with delusional disorder (Table 2). *ABCC9* encodes a subunit of an ATP-sensitive potassium channels. Functional estimation with SIFT, PROVEAN, and MutationTaster predicted that the missense mutations in *KIF26B* (Kinesin Family Member 26B) and *NAV3* (Neuron Navigator 3) in TT12 were disruptive to protein function (Supplementary Table S3).

One of the seven variants (chr1:245849059 C > T in TT12) were found in the Integrative Japanese Genome Variation Database (iJGVD 3.5KJPN)^34^ with an allele frequency of 0.0003. Three of the seven variants were found in the Exome Aggregation Consortium database (ExAC)^35^ but were exceptionally rare with an allele frequency range of 0.000008767–0.00004975. The missense variant in *ABCC9* was not found in neither database. Five of the seven TAS-validated HC sites (71.4%) were C > T transitions.

## Discussion

We successfully identified seven somatic mutations in blood samples derived from one pair of MZ twins discordant for delusional disorder. Five of the seven sites were further validated by pyrosequencing. Pyrosequencing could not validate two sites whose TAS-calculated AAFs were below 3%. We interpret this result as an outcome of the limited sensitivity of pyrosequencing^33^ and do not consider these variants to be false positives. Validation with a higher sensitivity is possible with digital droplet PCR, which is claimed to be able to detect mutations with allele fractions of 0.001%.^36^

Twenty-one of the 28 HC candidates were not validated by TAS. These false positives can plausibly be attributed to our relaxed filtering parameters. Depth of coverage was revealed to be one of the main parameters influencing sensitivity in selecting somatic mutation candidates from WES data. Compared to our previous results with whole genome sequencing,^37^ WES needed higher depth of 60× in detecting somatic mutations. The target enrichment process by biotinylated RNA library with reference-matched sequences has negative bias to non-reference alleles. This bias should necessitate higher depth in detecting non-reference alleles than whole genome sequencing, which has no target enrichment process. Alternatively, the false positives could have arisen owing to several other reasons: sample contamination, PCR-induced errors during library preparation, sequencing errors, and false alignment of the sequence reads.

Previous reports suggest that contamination during sample preparation or sequencing can indeed be a reason for the occurrence of false positives.^38,39^ We prevented sample contamination during sequencing by indexing, but contamination during preparation of genomic DNA for PCR procedures cannot be ruled out. Oxidative DNA damage is also reported to result in the occurrence of G > T and C > A false positives, derived from 8-oxoguanine, during library preparation.^40^ Since TAS-validated somatic mutations underwent two independent PCRs before sequencing (WES and TAS), they are unlikely to be false positives, but we cannot exclude their presence among the unvalidated HC candidates.

If the Phred-scale base quality is not always accurate, sequencing errors are also possible. HiSeq and MiSeq systems implement similar chemical reactions and adopt similar optical processes. Thus, similar systematic errors can arise within the sequencing systems resulting in the generation of false positives. If systematic sequencing errors generated false positives, the control samples should have exhibited commensurate false positive rates and at similar allele fractions. However, we observed a significant difference in the ratios of reference and alternate base-calls within the twin samples (Fischer’s exact test *p*-value < 2.2 × 10^−16^, Supplementary Table S4). Therefore, we assign a low probability to the occurrence of false positives due to systematic sequencing errors in our study. False alignment of homologous sequences is a common problem associated with the current short-read sequencing technologies. Therefore, we excluded regions having multiple homologous sequences in the reference genome by employing a BLAT score threshold of 160. However, a more stringent threshold could have increased the specificity of detection of HC candidate sites.

Based on the technical discussion above, we recommend a DP threshold > 60 and a more stringent BLAT threshold for future WES studies investigating somatic mutations. Coverage depth was the most influential factor affecting the selection of HC candidates through our filtering process. The depth is also a major factor determining sensitivity. The sensitivity of MuTect was estimated at 43.4% for detecting somatic mutations with an AAF of 3% at 82.2 × depth^41^ (average depth of our eight samples). In future, greater depth of sequencing could increase sensitivity, as well as specificity and would thus enable WES to detect somatic mutations in other MZ twins. The use of Strelka was not effective in increasing sensitivity, but it increased specificity when used in addition to MuTect in our analysis.

We can interpret the occurrence of somatic mutations in MZ twins in two ways. One interpretation is that these mutations occurred early in development and are therefore shared by the brain and other tissues. Indeed, somatic mutations have been found in relevant genes in the peripheral blood of patients with brain malformations^18,19^ and Rett syndrome,^20^ suggesting that it is highly probable that they are shared between brain and blood. In previous studies, the allele fractions of somatic mutations in the peripheral tissues of patients with brain malformations were reported to range between 1 and 43% for *PIK3CA*^18^ and between 5 and 35% for *DCX* and *LIS1*.^19^ The allele fractions of somatic mutations in blood samples ranged between 1.1 and 7.3% in our study. It is fair to hypothesize that the detected somatic mutations are shared between blood and brain in our study too, especially the missense mutation in *ABCC9* with an AAF of 7.32%.

Although the missense somatic mutations in *ABCC9* identified in TT11 diagnosed with delusional disorder was predicted as non-disruptive to protein function, *ABCC9* has been reported to be associated with sleep duration,^42^ and hippocampal sclerosis of aging (neuron loss, gliosis, and atrophy in the hippocampus).^43^ In addition, dominant missense mutations in *ABCC9* are known to cause Cantú syndrome, also known as hypertrichotic osteochondrodysplasia, which is characterized by congenital hypertrichosis, facial features, and cardiac defects.^44^ These studies suggest that the ATP-sensitive potassium channels containing mutated ABCC9 in brain may involve the pathophysiological electrical activity in neurons. Previous genetic analyses of patients with schizophrenia have found no de novo mutations in *ABCC9*,^25–31^ but three rare variants in patients with schizophrenia were reported in Genovese et al.^32^ Although not conclusive, somatic mutations in *ABCC9* might cause abnormal brain dysfunctions through potassium-dependent neural electricity, contributing to delusional disorder.

We detected two missense somatic mutations in *KIF26B* and *NAV*, both of which are expressed in neurons, in the co-twin with no psychiatric disorder (TT12). Although these mutations were predicted to disrupt protein function, this prediction cannot be reconciled with the fact that TT11, not TT12, had the psychiatric disorder. Thus, we cannot conclude definitively whether the identified somatic mutations contributed to the phenotypic discordance in our study.

An alternative explanation of our results can be that the blood stem cells bearing the somatic mutations underwent clonal expansion. Among the four pairs of MZ twins, somatic mutations were found only in the oldest pair, who were in their 60s. Older people tend to have a greater clonal expansion of blood stem cells, which can be detected by WES.^45,46^ One previous study implicated aging as one of the reasons for the occurrence of discordant mutations between MZ twins: it compared two pairs of MZ twins, one aged 40 and the other 100.^47^ The older pair in our study had no hematologic cancers, but we cannot rule out the influence of aging. Further studies involving other tissues will be needed to clarify whether somatic mutations are specific to blood samples.

We have demonstrated the existence of somatic mutations in blood samples obtained from MZ twins discordant for psychiatric disorders. Although the relationship between somatic mutations and the individual phenotype (delusional disorder in this study) remained unclear, we could identify somatic mutations with a high reliability by analyzing WES data derived from MZ twin samples. In theory, somatic mutations could contribute to the development of individual phenotypes in the form of rare variants of strong effects, or as additive variants with germline variants. Further studies on postmortem brain samples can clarify the pathophysiological role of somatic mutations in psychiatric disorders. In parallel, large cohorts of discordant MZ twins can clarify the association between somatic mutations and psychiatric disorders. Our approach for detecting somatic mutations with conventional WES data can be utilized for the genetic investigation of a large number of MZ twins.

## Methods

We designed and executed this study in accordance with the standards set by the Declaration of Helsinki (World Medical Association) and the guidelines provided by the Ministries of Education, Culture, Sports, Science and Technology and Health, Labour and Welfare of the Japanese government. The ethics committees of the University of Tokyo and the collaborating research institutions approved this study. The workflow is based on our previous study.^37^

### Sample procurement

Peripheral blood cells were derived from four Japanese female MZ twin pairs who were discordant for either schizophrenia or delusional disorder, diagnosed according to Diagnostic and Statistical Manual of Mental Disorders IV-TR (DSM-IV-TR; Table 1). Written informed consent was obtained from each participant.

### Whole exome sequencing

We performed WES of genomic DNA extracted from the eight samples. The DNA was extracted using Wizard Genomic DNA Purification Kit (Promega, Madison, WI, USA). Exons were enriched with SureSelect Human All Exon V4 and V5 + UTRs kits (Agilent Technologies, Santa Clara, CA, USA). The sequencing libraries were prepared with the TruSeq DNA HT Sample Prep Kits (Illumina, San Diego, CA, USA) including PCR procedures. We performed sequencing on the Illumina HiSeq 2000 platform, setting the read length to 100, and with indexing. HiSeq 2000 is designed to prevent carry-over (DNA contamination).

### QC and alignment

We performed QC and alignment according to the procedure described by DePristo et al.^48^ We merged all the raw sequence files belonging to the same individual into a single FASTQ file. The sequence data were quality controlled using FastQC (version 0.11.2) (http://www.bioinformatics.babraham.ac.uk/projects/fastqc/), and Trimmomatic (version 0.32).^49^ We stringently eliminated low-quality base calls to reduce false positives from the sequencing output, since we expected sequencing errors to be a major source of false positives. After QC, the sequence data were subjected to BWA (version 0.7.12)^50^ for alignment to the human reference genome (GRCh37) and a decoy genome (for reads that do not align to the reference genome) hosted by the Broad Institute (ftp://ftp.broadinstitute.org/). The alignment data (BAM files) were deduplicated by Picard (version 1.102) (https://github.com/broadinstitute/picard) and thereafter processed using GATK (version 3.2-2)^51^ for Indel realignment, and base recalibration, in that order. Only the data with mapping quality (mapQ) ≥ 1 as calculated by SAMtools (version 0.1.19)^52^ were selected. The analytical parameters were fine-tuned to optimize the final depth and base-call quality for somatic mutation detection (Supplementary Table S5).

The sequence data including depth and coverage are summarized in Table 1. For each pair of twins, monozygosity was assessed by comparing putatively credible SNV sites within the co-twins. The putatively credible SNV sites were defined by depth ranged between 80× and 90×, genotype quality of 99, and QUAL (quality score calculated by GATK) ≥ 1000 just for obtaining a rough estimate of monozygosity.

### Detection of somatic mutation candidates

We detected putative somatic mutations with the MuTect (version 1.1.5).^41^ We also used another software package Strelka (version 1.0.14)^53^ for additional analysis. We selected MuTect and Strelka for detecting somatic mutations because their sensitivity has been reported to be superior compared to other software packages.^54^

We ran MuTect and Strelka on the BAM files after QC. They called putative somatic mutations by comparing BAM files from the twin and the co-twin. Both the packages were operated with default parameters, except for isSkipDepthFilters, which was set to one for exome analysis in Strelka. The default parameters in MuTect and Strelka assume that the control sample has no somatic mutations identical to the target sample.

### Selection of somatic mutation candidates

We selected putative somatic SNVs according to our previous work.^37^

We excluded the genomic regions rich in sequences homologous to other regions, where accurate alignment is difficult with the current short read technology,^55^ as “multi-copy regions [MCRs]”. We defined the MCRs as “track: RepeatMasker, Interrupted Rpts (Interrupted Repeats), Microsatellite, Segmental Dups (Segmental Duplications), Self Chain, Simple Repeats” (1.63 Gb in total) in “group: Repeats” in the hg19 assembly available from the UCSC Genome Browser.^56^ We also excluded candidate sites ± 10 bp around indels (detected by UnifiedGenotyper in GATK-3.2-2) due to difficulty in accurate alignment.

We set the further filtering parameters as follows: average base quality of candidates (BQ) ≥ 20, coverage depth at a candidate site (DP) ≥ 30, second-highest score of UCSC BLAT using the sequence around a candidate site (±100 bp) < 160. The top score of the genomic region around a candidate site is always 200. The threshold of <160 excluded candidate sites that had homologous sequences in other genomic regions. The filtering parameters optimized for sensitivity for detecting SNVs. We stringently excluded candidates that were identical to candidates identified in the control samples, assuming that such sites were the result of sequencing errors and should be classified as false positives (MuTect and Strelka tolerate one or two base-calls of candidate mutations in control samples at default parameters, regarding those as sequence artifacts). Strelka computes an original score called Quality Score for Somatic SNV (QSS), in contrast to BQ computed by MuTect. For Strelka-called candidates, we adopted a threshold of QSS ≥ 20, analogous to BQ ≥ 20.

To identify reliable sequence reads containing somatic mutations, we defined Reliable Base-call supporting Somatic Mutation (RBSM).^37^ RBSM featured a somatic mutation candidate call with all the following features: (i) base quality ≥ 25, (ii) read mapping quality (mapQ) ≥ 30, (iii) not on the ±10 bp edge of read, (iv) not having minor mismatch or INDELs of ±15 bp on the same read, (v) no XA (alternative alignment) tags, (vi) not having soft-clip on the same read, (vii) not explained by INDEL realignment errors. We defined another term called difficult sequencing context (SeqContext) to mean the presence of short tandem repeat sites or poly-A sites. We then selected HC candidate sites, defined by an RBSM count ≥2 and the absence of a difficult SeqContext, upon visualization using IGV (version 2.3.40).^57^

### Target amplicon sequencing

We designed PCR primers for all the candidate sites and confirmed that they yielded single-banded amplicons of the expected sizes. Sequencing libraries were prepared by conducting two rounds of PCR. The libraries were sequenced in Illumina MiSeq using MiSeq Reagent Kit v3 at a theoretical depth of 200,000×. The primers used for TAS are listed in Supplementary Tables S6 and S7. The detailed protocol is described elsewhere in Nishioka et al.^37^

The TAS data were stringently quality controlled by Trimmomatic (version 0.32). After QC, we aligned the reads to the human reference genome (GRCh37) and a decoy reference genome by BWA (version 0.7.12). Thereafter we selected reads with mapQ ≥ 60. We then selected base-calls with base quality ≥ 20, and calculated an AAF for alternate base-calls with respect to the total base-calls at the candidate site. The candidates with an AAF > 0.316% (assumed sequence error rate, which is equivalent to base-call quality of 25) were considered as validated somatic mutations. For TAS-validated somatic mutations, functional annotation was performed with SnpEff (version 4.1).^58^ The effect of the mutation on protein function was estimated by SIFT (version 1.03),^59^ PROVEAN (version 1.1.3),^60^ and MutationTaster.^61^

### Pyrosequencing

TAS-validated somatic mutations were independently PCR-amplified using biotinylated primers. Briefly, genomic DNA (10 ng) was mixed with Q5 high-fidelity DNA polymerase, 200 μM of each dNTP, 2 mM MgCl_2_, and 0.5 μM of each primer (30 μl in total). The reaction mixture was subjected to PCR amplification under the following cycling conditions: 98 °C for 30 min; then, 33 cycles starting at 98 °C for 10 s, 64 °C for 10 s, and 72°C for 10 s; followed by 72 °C for 5 min. Annealing temperature was set to 64 °C to attain higher specificity than during TAS library preparation. The PCR product (30 μl) was mixed with 1.5 μl Streptavidin Sepharose High Performance medium (GE Healthcare, Chicago, IL, USA), 40 μl PyroMark Binding Buffer (Qiagen, Hilden, Germany), and 8.5 μl Milli-Q water, and shaken for 10 min. The resulting product was washed with 70% ethanol for 5 s, 0.2 N NaOH for 5 s, and 10 mM Tris–HCl (pH 7.6) for 10 s, and was then mixed with 38.5 μl PyroMark annealing buffer (Qiagen) and 1.5 μl sequence primer (10 μM). Finally, we performed pyrosequencing of the samples thus prepared by loading them into PyroMark Q96 (Qiagen) and operating it in the allele quantification (AQ) mode, according to the manufacturer’s protocol. Primers are listed in Supplementary Table S8.

### Statistical analysis

We performed *t*-test and Fischer’s exact test by R (version 3.2.3) (https://www.r-project.org/).

### Data availability

The WES and TAS data analyzed during the current study are not publicly available due to ethical regulations, but are available from the corresponding authors on reasonable request.

## Electronic supplementary material

Supplementary Tables S1 to S8
