# Supplementary material for: Identification of somatic mutations in monozygotic twins discordant for psychiatric disorders
Source: NPJ Schizophr. 2018 Apr 13;4:7. doi: 10.1038/s41537-018-0049-5 (PMC5899160; doi:10.1038/s41537-018-0049-5)
Supplement: Supplementary file 1 — Supplementary Tables S1 to S8 [file 41537_2018_49_MOESM1_ESM.docx]

**Supplementary Table S1. Summary of parameters employed for detecting and selecting somatic mutation candidates**

| **MuTect** | **MZ twin samples** | | | | | | | |
| --- | --- | --- | --- | --- | --- | --- | --- | --- |
| **Target** | FT11* | FT12 | JT11* | JT12 | TT21* | TT22 | TT11* | TT12 |
| **Control** | FT12 | FT11* | JT12 | JT11* | TT22 | TT21* | TT12 | TT11* |
| **MuTect** | 70 | 101 | 108 | 116 | 90 | 99 | 94 | 110 |
| **MCR filter** | 70 | 101 | 108 | 116 | 90 | 99 | 94 | 110 |
| **INDEL filter** | 60 | 96 | 102 | 111 | 83 | 92 | 89 | 104 |
| **AAF_ctrl = 0** | 38 | 66 | 64 | 70 | 58 | 68 | 53 | 63 |
| **BQ ≥ 20, DP ≥ 30** | 19 | 25 | 16 | 17 | 18 | 17 | 31 | 21 |
| **BLAT < 160** | 12 | 12 | 4 | 5 | 4 | 0 | 10 | 8 |
| **HC** | 3 | 7 | 1 | 2 | 3 | 0 | 6 | 6 |
| **Strelka** | **MZ twin samples** | | | | | | | |
| **Target** | FT11* | FT12 | JT11* | JT12 | TT21* | TT22 | TT11* | TT12 |
| **Control** | FT12 | FT11* | JT12 | JT11* | TT22 | TT21* | TT12 | TT11* |
| **Strelka** | 12 | 17 | 33 | 60 | 31 | 31 | 40 | 54 |
| **MCR filter** | 12 | 17 | 33 | 60 | 31 | 31 | 40 | 54 |
| **INDEL filter** | 10 | 17 | 31 | 57 | 31 | 31 | 39 | 45 |
| **AAF_ctrl = 0** | 9 | 11 | 23 | 45 | 24 | 23 | 33 | 32 |
| **QSS ≥ 20, DP ≥ 30 BLAT < 160** | 3 | 2 | 5 | 8 | 0 | 4 | 8 | 4 |
| **HC** | 1 | 1 | 3 | 5 | 0 | 2 | 6 | 4 |

MCR: Multi-Copy Region. INDEL: Insertion/Deletion. AAF_ctrl: Alternate Allele Fraction in control sample (%). BLAT: UCSC BLAT score. BQ: average Base Quality indicating somatic mutation. DP: DePth of coverage at target site. HC: High Confidence. QSS: Quality Score for Somatic SNV defined in Strelka. * indicates patient

**Supplementary Table S2. Alternate allele fractions of high-confidence somatic mutation candidates**

| **MZ twins** | |  |  |  |  |  | **Subject (WES)** | | | **Control (WES)** | | **Subject (TAS)** | | **Control (TAS)** | |  |
| --- | --- | --- | --- | --- | --- | --- | --- | --- | --- | --- | --- | --- | --- | --- | --- | --- |
| **Subject** | **Software** | **Chr** | **Position** | **Ref** | **Alt** | **BLAT** | **BQ** | **DP** | **AAF** | **DP** | **AAF** | **DP** | **AAF** | **DP** | **AAF** | **Results** |
| **FT11*** | **M** | 9 | 113341741 | C | T | NA | 33 | 46 | 6.5 | 40 | 0 | 143991 | 0.044 | 151078 | 0.037 | Not validated |
| **FT11*** | **M** | 19 | 49621802 | C | T | 23 | 34 | 45 | 6.7 | 63 | 0 | 181984 | 0.026 | 183934 | 0.012 | Not validated |
| **FT11*** | **M** | 21 | 31864375 | G | A | 55 | 33 | 47 | 6.4 | 48 | 0 | 184995 | 0.012 | 177311 | 0.017 | Not validated |
| **FT12** | **M** | 1 | 205242142 | C | A | 28 | 32 | 30 | 10.0 | 35 | 0 | 175596 | 0.008 | 187992 | 0.007 | Not validated |
| **FT12** | **M** | 7 | 142637430 | C | A | 24 | 33 | 40 | 7.5 | 40 | 0 | 168609 | 0.007 | 165539 | 0.007 | Not validated |
| **FT12** | **M** | 9 | 117014809 | G | A | 30 | 32 | 45 | 6.7 | 38 | 0 | 181804 | 0.023 | 160056 | 0.008 | Not validated |
| **FT12** | **M, S** | 9 | 139849022 | C | G | 45 | 33 | 78 | 7.8 | 75 | 0 | 44171 | 0.113 | 42346 | 0.085 | Not validated |
| **FT12** | **M** | 14 | 65560458 | G | A | 85 | 33 | 41 | 7.3 | 45 | 0 | 187233 | 0.054 | 180378 | 0.062 | Not validated |
| **FT12** | **M** | 16 | 75728247 | C | T | 29 | 33 | 50 | 6.0 | 47 | 0 | 177706 | 0.011 | 160548 | 0.016 | Not validated |
| **FT12** | **M** | 20 | 58411402 | C | T | 26 | 33 | 33 | 9.1 | 31 | 0 | 177480 | 0.026 | 175440 | 0.017 | Not validated |
| **JT11*** | **M** | 2 | 54871421 | A | G | 24 | 36 | 39 | 7.7 | 66 | 0 | 170480 | 0.008 | 148382 | 0.008 | Not validated |
| **JT12** | **M** | 7 | 29440490 | G | A | 26 | 32 | 60 | 6.7 | 59 | 0 | 175586 | 0.017 | 158943 | 0.011 | Not validated |
| **JT12** | **M** | 17 | 27381715 | C | A | 21 | 32 | 32 | 9.4 | 31 | 0 | 183143 | 0.037 | 154613 | 0.067 | Not validated |
| **TT21*** | **M** | 1 | 28607676 | G | A | 33 | 32 | 30 | 10.0 | 37 | 0 | 217106 | 0.014 | 215669 | 0.011 | Not validated |
| **TT21*** | **M** | 4 | 113110009 | G | A | 24 | 33 | 38 | 7.9 | 35 | 0 | 212589 | 0.011 | 222123 | 0.009 | Not validated |
| **TT21*** | **M** | X | 41077657 | G | A | 120 | 32 | 35 | 8.6 | 46 | 0 | 172232 | 0.012 | 169733 | 0.013 | Not validated |
| **TT11*** | **M** | 7 | 105641974 | G | T | 22 | 37 | 93 | 4.3 | 112 | 0 | 177376 | 2.417 | 180806 | 0.012 | Validated |
| **TT11*** | **M, S** | 11 | 72947061 | C | T | 23 | 35 | 65 | 9.2 | 77 | 0 | 188628 | 5.770 | 186399 | 0.010 | Validated |
| **TT11*** | **M, S** | 12 | 22040794 | A | C | 27 | 31 | 74 | 8.1 | 78 | 0 | 179669 | 7.320 | 182798 | 0.007 | Validated |
| **TT11*** | **M** | 1 | 52821154 | G | A | 26 | 32 | 33 | 9.1 | 39 | 0 | 185818 | 0.048 | 180707 | 0.063 | Not validated |
| **TT11*** | **M** | 12 | 42491817 | G | A | 92 | 34 | 46 | 6.5 | 63 | 0 | 212885 | 0.017 | 200265 | 0.017 | Not validated |
| **TT11*** | **M, S** | 19 | 44739117 | C | T | 24 | 34 | 73 | 5.5 | 73 | 0 | 191009 | 0.027 | 192628 | 0.019 | Not validated |
| **TT12** | **M** | 1 | 21605869 | G | A | 30 | 33 | 64 | 6.3 | 55 | 0 | 191426 | 3.830 | 191942 | 0.014 | Validated |
| **TT12** | **M, S** | 1 | 39991592 | C | T | 32 | 33 | 80 | 11.3 | 102 | 0 | 179781 | 6.588 | 173062 | 0.006 | Validated |
| **TT12** | **M, S** | 1 | 245849059 | C | T | 22 | 34 | 91 | 5.5 | 106 | 0 | 207113 | 1.120 | 204793 | 0.012 | Validated |
| **TT12** | **M, S** | 12 | 78571018 | C | T | 23 | 31 | 104 | 5.8 | 78 | 0 | 201424 | 3.092 | 196370 | 0.016 | Validated |
| **TT12** | **M** | 1 | 201010615 | G | A | 22 | 31 | 40 | 7.5 | 44 | 0 | 192446 | 0.006 | 193493 | 0.010 | Not validated |
| **TT12** | **M** | 14 | 64591770 | G | A | 66 | 34 | 47 | 6.4 | 52 | 0 | 185043 | 0.219 | 186252 | 0.213 | Not validated |
| **FT11*** | **S** | 9 | 116779003 | C | T | NA | (35) | 141 | 4.3 | 151 | 0 | 180142 | 0.024 | 195770 | 0.021 | Not validated |
| **JT11*** | **S** | 2 | 175664545 | G | A | 140 | (22) | 46 | 8.7 | 73 | 0 | 164701 | 0.044 | 138271 | 0.012 | Not validated |
| **JT11*** | **S** | 7 | 48413830 | G | A | 20 | (22) | 56 | 7.1 | 58 | 0 | 187957 | 0.015 | 166505 | 0.019 | Not validated |
| **JT11*** | **S** | 22 | 43253229 | G | A | 33 | (22) | 43 | 9.3 | 48 | 0 | 119623 | 0.011 | 100607 | 0.010 | Not validated |
| **JT12** | **S** | 2 | 114718299 | G | A | 30 | (23) | 53 | 7.5 | 56 | 0 | 189889 | 0.008 | 156027 | 0.006 | Not validated |
| **JT12** | **S** | 3 | 42252628 | T | C | 22 | (20) | 54 | 7.4 | 41 | 0 | 186149 | 0.010 | 158963 | 0.007 | Not validated |
| **JT12** | **S** | 12 | 123752524 | G | T | 22 | (20) | 34 | 11.8 | 39 | 0 | 180015 | 0.007 | 151686 | 0.005 | Not validated |
| **JT12** | **S** | 19 | 42352925 | G | A | 32 | (27) | 31 | 12.9 | 51 | 0 | 172060 | 0.006 | 211555 | 0.010 | Not validated |
| **JT12** | **S** | X | 37587307 | C | A | 26 | (23) | 166 | 3.6 | 119 | 0 | 180906 | 0.003 | 163703 | 0.003 | Not validated |
| **TT22** | **S** | 1 | 28833911 | C | A | 117 | (21) | 48 | 8.3 | 44 | 0 | 208262 | 0.011 | 205429 | 0.009 | Not validated |
| **TT22** | **S** | 12 | 54070004 | G | A | NA | (20) | 114 | 4.4 | 122 | 0 | 179211 | 0.012 | 175962 | 0.013 | Not validated |
| **TT11*** | **S** | 1 | 78603073 | G | A | 24 | (22) | 53 | 7.5 | 86 | 0 | 192305 | 0.014 | 205610 | 0.010 | Not validated |
| **TT11*** | **S** | 10 | 50960631 | T | C | 27 | (25) | 48 | 8.3 | 55 | 0 | 196603 | 0.008 | 198006 | 0.006 | Not validated |
| **TT11*** | **S** | 19 | 5208010 | G | A | 23 | (26) | 106 | 4.7 | 109 | 0 | 182979 | 0.028 | 178617 | 0.070 | Not validated |
| **TT12** | **S** | X | 50119242 | G | A | 26 | (20) | 41 | 9.8 | 40 | 0 | 169088 | 0.005 | 190112 | 0.007 | Not validated |

Software: M = Mutect, S = Strelka. BLAT: UCSC BLAT score. BQ: average Base Quality indicating somatic mutation. (parenthesis) means QSS (Quality Score for Somatic SNV) in Strelka. DP: DePth of coverage at target site. AAF: Alternate Allele Fraction, indicating somatic mutation (%). NA: Not Available. * indicates patient. The genomic positions are based on the reference genome GRCh37.

**Supplementary Table S3. Known allele frequencies and predicted functional effects of the validated mutations**

| **Subject** | **Chr** | **Position** | **Ref** | **Alt** | **Gene** | **SO** | **HGVS.p** | **Functional Estimation** | | | **Allele frequency in Database** | |
| --- | --- | --- | --- | --- | --- | --- | --- | --- | --- | --- | --- | --- |
|  |  |  |  |  |  |  |  | **SHIFT** | **PROVEAN** | **MutationTaster** | **ExAC** | **iJGVD (3.5KJPN)** |
|  |  |  |  |  |  |  |  |  |  |  |  |  |
| **TT11*** | 7 | 105641974 | G | T | *CDHR3* | synonymous_variant | p.Ala260Ala | tolerated | neutral | polymorphism | none | none |
| **TT11*** | 11 | 72947061 | C | T | *P2RY2* | 3_prime_UTR_variant |  |  |  |  | none | none |
| **TT11*** | 12 | 22040794 | A | C | *ABCC9* | missense_variant | p.Leu626Arg | tolerated | neutral | polymorphism | none | none |
| **TT12** | 1 | 21605869 | G | A | *ECE1* | missense_variant | p.Pro20Leu | tolerated | neutral | polymorphism | 0.000008767 (1/114058) | none |
| **TT12** | 1 | 39991592 | C | T | *BMP8A* | 3_prime_UTR_variant |  |  |  |  | none | none |
| **TT12** | 1 | 245849059 | C | T | *KIF26B* | missense_variant | p.Thr925Met | damaging | deleterious | disease causing | 0.00003481 (4/114906) | 0.0003 (2/7094) |
| **TT12** | 12 | 78571018 | C | T | *NAV3* | missense_variant | p.Pro1741Leu | damaging | deleterious | disease causing | 0.00004975 (6/120602) | none |

SO: Sequence ontology categories defined by the Sequence Ontology project (<http://www.sequenceontology.org/>). HGVS.p: Amino acid change described according to the Human Genome Variation Society. ExAC: The Exome Aggregation Consortium (accessed on August 9, 2017). iJGVD: Integrative Japanese Genome Variation Database (accessed on October 1, 2017). * indicates patient (with delusional disorder in this table). The genomic positions are based on the reference GRCh37

**Supplementary Table S4. Fisher’s exact test comparing reference and alternate basecalls within MZ twin pairs (TAS)**

| **Chr** | **Position** | **Ref** | **Alt** | **TT11*** |  |  | **TT12** |  |  |  |
| --- | --- | --- | --- | --- | --- | --- | --- | --- | --- | --- |
|  |  |  |  | **Ref basecall** | **Alt basecall** | **AAF** | **Ref basecall** | **Alt basecall** | **AAF** | **Fisher's exact test p-value** |
| 7 | 105641974 | G | T | 173060 | 4287 | 2.417 | 180763 | 21 | 0.012 | < 2.2 ×10^-16^ |
| 11 | 72947061 | C | T | 177734 | 10883 | 5.770 | 186358 | 19 | 0.010 | < 2.2 ×10^-16^ |
| 12 | 22040794 | A | C | 166489 | 13152 | 7.320 | 182761 | 12 | 0.007 | < 2.2 ×10^-16^ |
| 1 | 21605869 | G | A | 191904 | 26 | 0.014 | 184056 | 7332 | 3.830 | < 2.2 ×10^-16^ |
| 1 | 39991592 | C | T | 173046 | 10 | 0.006 | 167928 | 11844 | 6.588 | < 2.2 ×10^-16^ |
| 1 | 245849059 | C | T | 204763 | 25 | 0.012 | 204787 | 2319 | 1.120 | < 2.2 ×10^-16^ |
| 12 | 78571018 | C | T | 196319 | 32 | 0.016 | 195166 | 6229 | 3.092 | < 2.2 ×10^-16^ |

AAF: Alternate Allele Fraction, indicating somatic mutation (%). * indicates patient. The genomic positions are based on the reference genome GRCh37.

**Supplementary Table S5. Settings of software parameters adopted in our analysis pipeline**

| **Software** | **Parameter** | **Value** |
| --- | --- | --- |
| Trimmomatic | ADAPTOR | TruSeq3-PE.fa:2:30:10 (Illumina Adaptor Sequence) |
|  | TRAILING | 5 |
|  | LEADING | - |
|  | SLIDINGWINDOW |  |
|  | MINLEN | 30 |
| BWA | reference | NCBI build37 + decoy (Broad Institute) |
| Picard Deduplication |  | yes |
| GATK RealignerTargetCreator IndelRealigner | known | 1000G_phase1.indels.b37.vcf (Broad Institute) Mills_and_1000G_gold_standard.indels.b37.vcf (Broad Institute) |
|  | targetIntervals | each bam files (each lane) |
|  | mode | USE_READS |
|  | LOD | 1.0 |
| Picard FixMateInformation |  | yes |
| GATK BaseRecalibrator PrintReads | known_sites | dbsnp_138.b37.vcf 1000G_phase1.indels.b37.vcf Mills_and_1000G_gold_standard.indels.b37.vcf (Broad Institute) |
| Samtools | q (mapQ) | 1 |

**Supplementary Table S6. PCR primers used during TAS library preparation**

| **Primer_name** | **Sequence** |
| --- | --- |
| SeqPrimer_F | TCTTTCCCTACACGACGCTCTTCCGATCT |
| SeqPrimer_R | GTGACTGGAGTTCAGACGTGTGCTCTTCCGATCT |
| TruSeq_F | AATGATACGGCGACCACCGAGATCTACACTCTTTCCCTACACGACGCTCTTCCGATCT |
| TruSeq_R_idxA001 | CAAGCAGAAGACGGCATACGAGATCGTGATGTGACTGGAGTTCAGACGTGTGCTCTTCCGATCT |
| TruSeq_R_idxA002 | CAAGCAGAAGACGGCATACGAGATACATCGGTGACTGGAGTTCAGACGTGTGCTCTTCCGATCT |
| TruSeq_R_idxA003 | CAAGCAGAAGACGGCATACGAGATGCCTAAGTGACTGGAGTTCAGACGTGTGCTCTTCCGATCT |
| TruSeq_R_idxA004 | CAAGCAGAAGACGGCATACGAGATTGGTCAGTGACTGGAGTTCAGACGTGTGCTCTTCCGATCT |
| TruSeq_R_idxA005 | CAAGCAGAAGACGGCATACGAGATCACTGTGTGACTGGAGTTCAGACGTGTGCTCTTCCGATCT |
| TruSeq_R_idxA006 | CAAGCAGAAGACGGCATACGAGATATTGGCGTGACTGGAGTTCAGACGTGTGCTCTTCCGATCT |
| TruSeq_R_idxA007 | CAAGCAGAAGACGGCATACGAGATGATCTGGTGACTGGAGTTCAGACGTGTGCTCTTCCGATCT |
| TruSeq_R_idxA008 | CAAGCAGAAGACGGCATACGAGATTCAAGTGTGACTGGAGTTCAGACGTGTGCTCTTCCGATCT |

**Supplementary Table S7. Primers used for TAS validation of each genomic site**

| **Chr** | **Position** | **Forward Primer** | **Reverse Primer** | **Length (bp)** |
| --- | --- | --- | --- | --- |
| 1 | 21605869 | TCACCTGCAGGGAAGGAG | TGCTTGACTCTCTGATGTTTGG | 226 |
| 1 | 28607676 | GGTGAATGGCAAGTAGGAGGTA | ACTTGGAGATCCTAAGGGACCA | 247 |
| 1 | 28833911 | TGTGGAAAGGGACTTGTACATC | TTTTTAACACCCCACTGTGGAC | 226 |
| 1 | 39991592 | ACAGCTCAAGCAGGAGTGTCA | CCACAGTCTGACAGGTCCTAAG | 222 |
| 1 | 52821154 | CCTGGAGAGTTTGTGTTCTTCA | AACTCGGTGAGGTTCAGGTAGA | 217 |
| 1 | 78603073 | CCAAAAAGGAAGGAGAAACTGA | AGTGAGTTCGTTTTGGAGGTTC | 230 |
| 1 | 201010615 | AAGAGACAGAGACGCCTGCTAC | GTAGCTGGTCCTGATGGTTTTC | 229 |
| 1 | 205242142 | AGTGTCTTCCCTGCTGAGTACC | ACAACCTCCTCCACACGAATAC | 225 |
| 1 | 245849059 | GCAAGTCAGAAAGGGACTGC | TTCGGACCCATTATCTTCCTTA | 215 |
| 2 | 54871421 | GTTTCTCCTTCACCACAGCTTC | GGGGTCGTTGTTGATTTTATTG | 222 |
| 2 | 114718299 | CTTTTCCCCCTTTTCTTACCAG | AGAACATTTTGCTCCCAGAGTC | 211 |
| 2 | 175664545 | CATAGACCCCAAAAGCAAAGTC | AACAGTGAACACACGCTTTCTG | 223 |
| 3 | 42252628 | CTTTCCTGCCCTGTATTTCAAG | TGCTCTGTCTAGACTGGACCTC | 220 |
| 4 | 113110009 | GCTCTGGTTCCACCAGTACCTA | TTTTTCCCATATCAAAGCCAAA | 214 |
| 7 | 29440490 | CCTATAGTTAACCCGCCTGTTG | ACAAAAGCTGCCGAGTACATTT | 236 |
| 7 | 48413830 | TAGTGTTTTGATTGGGACCACA | GACACTTCAAGCATCTCGGTAA | 226 |
| 7 | 105641974 | ACAGTCCTGGAGGAACTGAGTC | ACCAATTCTCATGGGTAACGAG | 229 |
| 7 | 142637430 | CTAGGCTTCAACGTGTTTTTCC | TGGAAAGAGTGCTTGTGAACAG | 225 |
| 9 | 113341741 | CGACCTTTCAGCAGATGTCC | AATCATCCACCAGGAAGACAAG | 216 |
| 9 | 116779003 | CAGAAACTGGCAATTACACCTG | AAAGACTGACCCAGAACTGAGC | 224 |
| 9 | 117014809 | AGGGACCCTCTAGAGACCTTGA | GGCAAGAGGATGCAGACTTACT | 214 |
| 9 | 139849022 | GGACCCAGAGGGAGGAGA | GAGTTTGGTTGACCCTAGAGGA | 349 |
| 10 | 50960631 | AAACTGGGTAAGACCCTTCCTC | CTCTCAGGCTTGAACACATGG | 224 |
| 11 | 66307068 | GCCAGCACTCCTATGACCTG | ACCCGACTTCCTTACTGAGTCT | 239 |
| 11 | 72947061 | GACAGCTAGTGAGAAGGCAGGT | AAGAGATGAACATCTGGGGACT | 225 |
| 12 | 22040794 | AGCACTTACAACTCCAGTGTGC | CACAGGCATCCTACTCACCATA | 230 |
| 12 | 42491817 | GATCACCCCATGTGATGTTTAG | TTGTGAGGAGTTTGCATTTCTC | 214 |
| 12 | 54070004 | CCCTGTAGTTTCTCCTCTCGAA | CCCACTGCAGAGAAGACAGAG | 212 |
| 12 | 78571018 | CAACTTACGCTGAAGCAGATTG | CCAAGTGCAAATAGACAAGAAGG | 226 |
| 12 | 123752524 | AAGGGCTCTGTATCCCAAGAC | GTTACCGCTGTTTGTCATTCTG | 224 |
| 14 | 64591770 | AATTAACATCAGCAGGGTCTAAA | CACATTGTGTGCAGCTTCTACC | 210 |
| 14 | 65560458 | TCAGGAAACTCACCTTCTCTCC | ACAGTGCTGACAGCGTGTACTT | 220 |
| 16 | 75728247 | TTTTCTTGCTCACATTCTTCCA | CTTTGTCAGAATTGGCAGTGTC | 210 |
| 17 | 27381715 | ACTGGCATCTACCTGCCTTATC | TCTTCATTTCCCACTTCCTCTC | 219 |
| 19 | 5208010 | TAGCATCTTCACCGTCTGAAAG | AAGCTTAGGCTGTCCCATCTG | 214 |
| 19 | 42352925 | GAGACAGCAGGTTCTTGGTACA | CCGTATCCCAGAATACACTCAA | 237 |
| 19 | 44739117 | TTTGCAGGTTATTTTTCACATCA | AGGTGTCTTCAGGGGAAGAGTT | 223 |
| 19 | 49621802 | TCTTGAGATGTGGAGGGAATG | CTCAGACGCTCTTAAGGGTAGG | 230 |
| 20 | 58411402 | AGGAGAGAAAGTGAACCTTTGG | ACAGATCTCCTCGCTGCTTAAA | 218 |
| 21 | 31864375 | TAGTGAGTTTGGTTTGCTGCTC | TGTTAAAGGGACAGCATGAGAA | 229 |
| 22 | 43253229 | AGATGGTCAAGATGTCCTGCTT | AAACGTTGGTGGATCCTATGG | 395 |
| X | 37587307 | ATCAACATGTTCTGCTGGTCTG | TGAAGAGAATGGCTGTGCAGTA | 229 |
| X | 41077657 | ATGCATTGTGTATTCTCCTTCG | TGACTAGAAGGTCCAGGAGAGG | 220 |
| X | 50119242 | GCAAGGACAGATAGATGAGTTGG | GGTGAGACAAGGATGGGTAAAG | 213 |

The genomic positions are based on the reference genome GRCh37.

**Supplementary Table S8. Primers used during pyrosequencing**

| **Chr** | **Position** | **PCR Primer1** | **PCR Primer2 (biotinylated)** | **Pyrosequence Primer** |
| --- | --- | --- | --- | --- |
| 1 | 21605869 | TCACCTGCAGGGAAGGAG | TGCTTGACTCTCTGATGTTTGG | GCGGGGAAGACGTGAGCCCC |
| 1 | 39991592 | ACAGCTCAAGCAGGAGTGTCA | CCACAGTCTGACAGGTCCTAAG | AGGGGCCCTCACTCTCGGTG |
| 1 | 245849059 | GCAAGTCAGAAAGGGACTGC | TTCGGACCCATTATCTTCCTTA | GGACTGCCTGAAGTGCAACA |
| 7 | 105641974 | ACAGTCCTGGAGGAACTGAGTC | ACCAATTCTCATGGGTAACGAG | ATCGTGGCCAATATCACAGC |
| 11 | 72947061 | AAGAGATGAACATCTGGGGACT | GACAGCTAGTGAGAAGGCAGGT | CAAGGGTCCTTTCTCCAATC |
| 12 | 22040794 | AGCACTTACAACTCCAGTGTGC | CACAGGCATCCTACTCACCATA | TCTTACAGGACTCAAAAGGA |
| 12 | 78571018 | CCAAGTGCAAATAGACAAGAAGG | CAACTTACGCTGAAGCAGATTG | GCTTACTGATTCATCCCTTC |

The genomic positions are based on the reference genome GRCh37.
